# Embedding Carbon Dots in Superabsorbent Polymers for Additive Manufacturing

**DOI:** 10.3390/polym10080921

**Published:** 2018-08-17

**Authors:** Yiqun Zhou, Keenan J. Mintz, Cagri Y. Oztan, Sajini D. Hettiarachchi, Zhili Peng, Elif S. Seven, Piumi Y. Liyanage, Sabrina De La Torre, Emrah Celik, Roger M. Leblanc

**Affiliations:** 1 Department of Chemistry, University of Miami, Coral Gables, FL 33146, USA; YXZ431@miami.edu (Y.Z.); kjm154@miami.edu (K.J.M.); sdh86@miami.edu (S.D.H.); z.peng@umiami.edu (Z.P.); ess133@miami.edu (E.S.S.); pyl5@miami.edu (P.Y.L.); 2 Department of Aerospace and Mechanical Engineering, University of Miami, Coral Gables, FL 33146, USA; cyo4@miami.edu (C.Y.O.); e.celik@miami.edu (E.C.); 3 Department of Biology, Florida International University, Miami, FL 33199, USA; sdela065@fiu.edu

**Keywords:** carbon dots, solvent effect, embedment, superabsorbent polymer, additive manufacturing, 3D printing

## Abstract

A type of orange carbon dots (O-CDs) synthesized via an ultrasonication route with citric acid and 1,2-phenylenediamine as precursors was embedded into sodium polyacrylate (SPA) as the ink for 3D printing. Characterizations of these spherical O-CDs revealed an ultra-small size (~2 nm) and excitation-independent, but solvent dependent, emission. The O-CDs were evenly distributed with low degree of aggregation in sodium polyacrylate (SPA), which was achieved due to the property that SPA can absorb water together with O-CDs. The 3D printed photoluminescent objective with the ink revealed a great potential for high yield application of these materials for additive manufacturing. This also represents the first time, bare CDs have been reported as a photoluminescent material in 3D printing, as well as the first time SPA has been reported as a material for 3D printing.

## 1. Introduction

Additive manufacturing (3D printing) has gained much attention recently due to the ability to produce complex figures and shapes and create rapid prototypes [1]. Printing in three dimensions is accomplished by depositing layer on layer to form the desired object [2]. It has recently gained interest in the manufacturing of normal objects such as eyeglasses and figurines, but also in producing biologically significant objects such as tissue and organs [3,4]. In addition, 3D printing technique has been widely applied in the manufacturing and study of optics and plasmonics in recent years [5,6]. Therefore, the enormous capability of 3D printing suggests the need for new materials with unique properties. Fluorescent materials could help to expand the applications of 3D printing, particularly in biological fields where the tracking of the material would be highly advantageous [7]. In order to make these materials effective they should be highly photostable and biocompatible. Common organic dyes and fluorophores do not meet these criteria; however carbon dots (CDs) show particular promise.

Carbon dots (CDs) have attracted growing interests recently because of their well-dispersion in water, nontoxicity and biocompatibility [8]. Synthetic methods can be categorized as “top-down” and “bottom-up” approaches [9]. Based on the selection of synthetic approaches, the starting materials and reaction conditions vary significantly [10,11]. Photoluminescence (PL), an important optical property of CDs, has often been used to characterize the formation of CDs after synthetic reactions. Quantum yield (QY), as a measurement of PL, has reached as high as 93.3% in 2015 [12] resulting from concerted efforts to improve synthetic approaches and surface modification, namely passivation and functionalization of CDs [13]. Therefore, the improvement of QY is no longer a farseeing goal of CDs synthesis.

Furthermore, most CDs exhibit blue or green PL with excitation-dependent PL behaviors [14,15], which is disadvantageous for many biological applications due to the presence of natural autofluorescence in some biological tissues [16]. Also, many nanoparticles such as lanthanide doped NaYF_4_ have been reported to be fluorescent in the longer excitation wavelength even near infrared (IR), which exhibited great potential in the application of security printing and invisible inks [17,18]. Therefore, the focus of CDs research should be directed towards the synthesis of long-wavelength-emissive, excitation-independent CDs as well as their biological applications and printing. However, low fabrication yield of CDs and their low QY in solid state are another two parameters limiting their applications in the 3D printing and manufacturing [19]. Therefore, it is necessary to amplify the performance of CDs in terms of PL in small amounts and in the solid state.

Herein, a solution is introduced by embedding a type of orange CDs (O-CDs), which exhibited the orange color in aqueous solution, in sodium polyacrylate (SPA) (with crosslinker), a superabsorbent polymer [20] to obtain solid state fluorescence. SPA has been found by the authors to absorb O-CDs in aqueous solution along with water. As the O-CDs can be well dispersed in water, they can easily be absorbed during the hydration (swelling) of SPA which results in an even distribution of O-CDs in polymer matrix (confirmed with transmission electron microscopy (TEM)). Possible hydrogen bonds between the surface functional groups of O-CDs and the polymer chains provide immobilization of O-CDs in the polymer matrix and stabilize the composite material [21]. Because of these interactions and/or physical entanglement, O-CDs are likely to stay in the polymer matrix even after the desorption of water. It is worth noting that CDs-SPA composite exhibited a strong PL even in solid-state. Therefore, CDs-SPA nanocomposite is an excellent material to achieve the amplification of the QY of O-CDs in solid state. Also, the use of CDs-SPA nanocomposite rather than unbound O-CDs will allow for novel applications which require high amounts of photoluminescent nanomaterials such as 3D printing.

Up to now, SPA has not been reported as a material for 3D printing. The use of SPA as a means of embedding fluorescent materials in a photopolymer will greatly increase the ease of production of fluorescent materials. Additionally, CDs have also not been reported as a photoluminescent additive for 3D printing. However, the ability of SPA to incorporate the fluorescent marker (CDs) has been demonstrated and the fluorescence in the solid state is clearly shown. Also, the use of CDs for 3D printing has great potential over other fluorescent species due to their high photostability [22,23]. The combination of these two materials will facilitate the production of fluorescent materials and further expand the applications of 3D printed materials.

## 2. Materials and Methods

### 2.1. Materials

Citric acid (99.5–100%) was purchased from VWR (West Chester, PA, USA). 1,2-phenylenediamine flakes (99.5%) and cross-linked sodium polyacrylate (SPA) were obtained from Sigma-Aldrich (St. Louis, MO, USA). The commercial FLGPCL02 photopolymer was bought from formlabs (Somerville, MA, USA) with a viscosity of between 850 and 900 cps and a data sheet containing its physical properties in the supporting information. The Orbeez commercial SPA-based beads were provided by Toys“R”Us (Miami, FL, USA) with a data sheet in the supporting information. The distilled water used was purified using a Modulab 2020 water purification system acquired from Continental Water System Corporation (San Antonio, TX, USA). The water had a pH of 6.62 ± 0.3 at 25 ± 0.5 °C. The SEC was performed using GE Healthcare Sephacryl S-300 (Uppsala, Sweden) as the matrix. All the chemicals were used without further treatment.

### 2.2. Instruments

The UV/vis absorption spectrum of O-CDs was obtained by using a Cary 100 UV/vis spectrophotometer (Santa Clara, CA, USA) by using a 1 cm optical cell. A Fluorolog (Horiba Jobin Yvon) spectrometer (Irvine, CA, USA) was used to record the fluorescence emission spectra of sample by using a slit width of 5 nm for both excitation and emission. As for the determination of fluorescence quantum yield, a Varian Cary Eclipse spectrometer (Santa Clara, CA, USA) was used to record the fluorescence spectra of samples and standards. Fourier-transform infrared (FTIR) spectroscopy data were obtained with a PerkinElmer FTIR (Frontier) spectrometer (Waltham, MA, USA) by using the attenuated total reflection (ATR) technique with air as background. The Zeta potential measurements were made by using a nano series Malvern Zetasizer (Westborough, MA, USA). AFM images of O-CDs were obtained with an Agilent 5420 atomic force microscope (Santa Clara, CA, USA) by using the tapping mode. To perform AFM measurement, a drop of diluted O-CDs aqueous solution was applied on a clean silica mica slide and air dried, which then was transferred to do the screening using tapping mode. As for the tip, we used silicon tips (length: 225 µm; thickness: 5 µm) manufactured from Nanosensors with a force constant of 15 N/m. TEM was performed by using a JEOL 1200X TEM (Peabody, MA, USA). For TEM measurements, a drop of the O-CDs solution was placed on a carbon coated copper grid and air dried prior to examination.

### 2.3. O-CDs Synthesis

Synthesis of O-CDs involved the use of 0.02 g citric acid as the carbon source and 0.28 g 1,2-phenylenediamine as the N-dopant with a molar ratio of 1:25 dissolved in 10 mL deionized H_2_O. In an ultrasonication bath the mixture was then sonicated for 1 h at a frequency of 42 kHz under the protection of argon gas. An orange solution was obtained showing yellow emission under a UV lamp (365 nm). After filtration of the unreacted 1,2-phenylenediamine in the ice bath and removal of small fluorophores by size exclusion chromatography (SEC), O-CDs remained in aqueous solution. After evaporation of water, O-CDs were obtained as a brown powder.

### 2.4. Embedment of O-CDs into SPA Powder as Feedstock for 3D Printing

First, 200 g of SPA powder was mixed with 0.8 L of 0.5 g/L O-CDs aqueous solution. After stirring for 5 min, the mixture became gelatinous. Subsequently, the gel was heated overnight. In order to attain the highest possible homogeneity, O-CDs embedded-SPA powder was crushed in a high-speed blender (blade speed >20,000 rpm) for 5 min and the crushed powders were input into 600-micron sieve to eliminate particles above this size. Then the refined powders could be used as the feedstock for 3D printing.

### 2.5. 3D Printing of the CDs-SPA Composite

The 3D printing process involved SLA (stereolithography) as the method of fabrication. Specimens were printed in a Formlabs 3D printer with ultimate quality selected on PreForm software (Somerville, MA, USA). The CDs-SPA composite was then mixed with 150 mL commercial FLGPCL02 photopolymer resin at a ratio of 25% by mass and filled inside the tank of the printer. In addition, layer height and printing speed were maintained at 0.025 mm and 3 cm/h, respectively and other parameters of printer are provided in the supporting information. Printed specimens were then subjected to post-curing under a commercial curing UV light source (MelodySusie 36W UV Nail Dryer, Union City, CA, USA) with a wavelength of 365 nm for 2 h to ensure proper hardening.

## 3. Results and Discussion

### 3.1. Characterization of O-CDs

**Figure 1:**
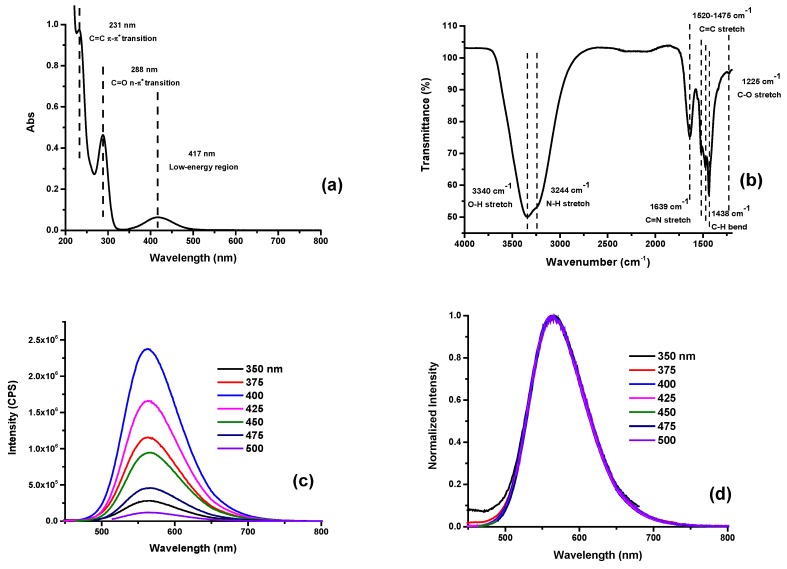
(**a**) The UV/vis absorption and (**b**) The attenuated total reflection-Fourier-transform infrared (ATR-FTIR) spectrum of orange carbon dots (O-CDs) powder; (**c**,**d**) fluorescence spectrum and the normalized fluorescence spectrum of O-CDs aqueous solution. The concentration of O-CDs for UV/vis and fluorescence measurement is 0.03 and 0.005 mg/mL, respectively.

The prepared O-CDs were characterized by spectroscopic measurements such as UV/vis, fluorescence emission and ATR-FTIR spectroscopies. In the UV/vis absorption spectrum (Figure 1a), C=C and C=O π–π* transition peaks are clearly shown at 231 and 288 nm, respectively. Another peak located in the lower-energy region at 417 nm is observed, which could be attributed to the absorption cross section of NO_2_ [24]. ATR-FTIR spectrum with accumulation of 6 scans (Figure 1b) records the functional groups of O-CDs consist of O–H (3340 cm^−1^) and N–H (3244 cm^−1^) [25,26], which contributes to the high water-dispersity, and C=C (1520–1475 cm^−1^), C–H (1438 cm^−1^) with C–O (1225 cm^−1^) [25] come from the starting materials. Unlike most CDs previously reported, O-CDs exhibit excitation-independent PL behavior, which indicates that the emission doesn’t shift with the change of excitation wavelength (Figure 1c,d). It can be ascribed to the single surface state caused by the uniform functional composition on the surface of CDs [27,28]. From the fluorescence emission spectrum, we observe that maximum excitation wavelength is 400 nm while the corresponding emission wavelength is 570 nm, which is the source of yellow fluorescence. As for the QY, tris(bipyridine)ruthenium(II) chloride in H_2_O (2.8%, QY) was used as the standard and the QY of O-CDs was calculated as 1% (see the supporting information for details of calculation). The low QY could be possibly due to the fluorescence quenching caused by the self-aggregation [29], which has been confirmed by the Zeta potential, a value to determine the strength of repulsion force between individual O-CDs particles.

**Figure 2:**
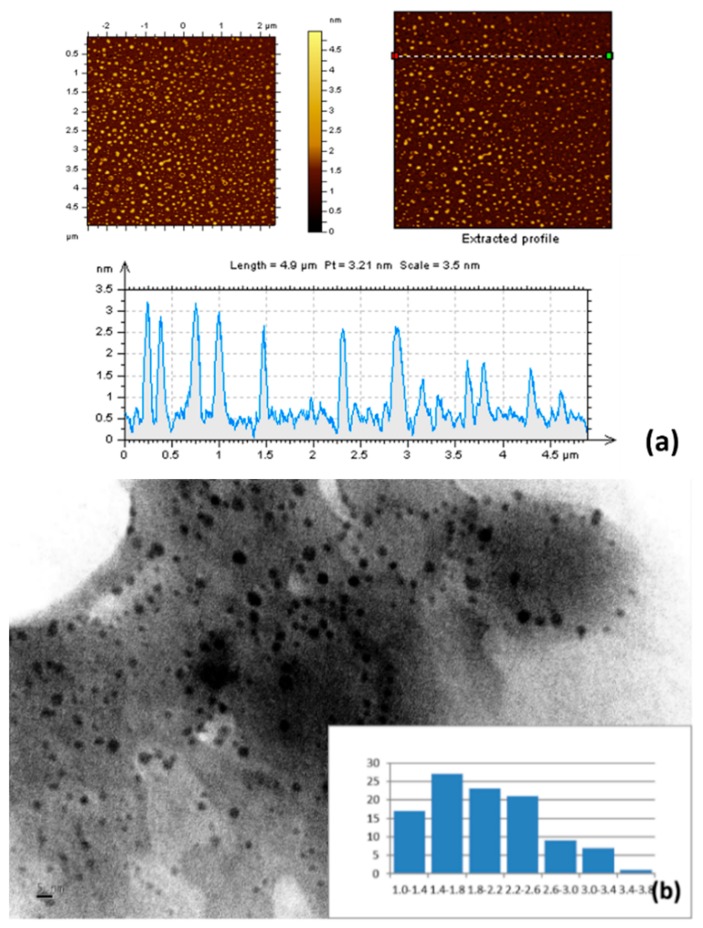
(**a**) Atomic force microscopy (AFM) and (**b**) transmission electron microscopy (TEM) images (inset: histogram of the size) of O-CDs.

The prepared O-CDs are small, which is reflected in the atomic force microscopy (AFM) and TEM results. In AFM images (Figure 2a), we measured the average height of O-CDs as 2 nm. As shown in the size histogram plot obtained from the TEM images (Figure 2b), the size of O-CDs is narrowly distributed within 1–4 nm with a mean diameter of 2 nm, which is consistent with the AFM data. Therefore, O-CDs are spherical in shape with a mean diameter of 2 nm. In addition, the Zeta potential was measured by a nano series Malvern Zetasizer. It indicated O-CDs carried a negative charge with a potential value of −12.2 mV, which revealed a weak repulsion interaction among O-CDs nanoparticles and led to the self-aggregation.

### 3.2. Solvent Effect of O-CDs

**Figure 3:**
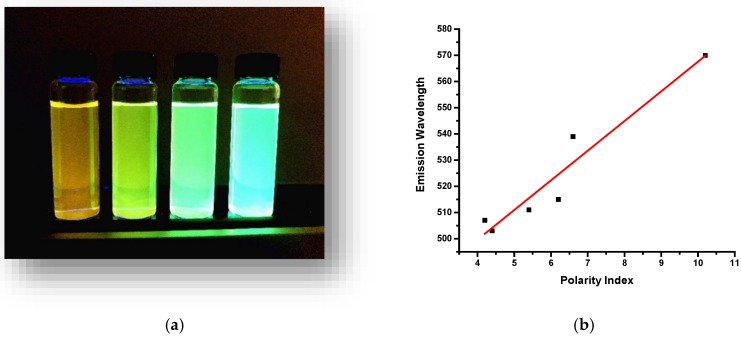
(**a**) Solvent effect of O-CDs (0.15 mg/mL). (Solvent from left to right: water, methanol, acetone and tetrahydrofuran). (**b**) X axis: the polarity index of different solvents (water, methanol, acetonitrile, acetone, chloroform, tetrahydrofuran); Y axis: the maximum emission wavelength of O-CDs; the linear relation between the two factors can be described as following equation: Y = 11.33X + 454.31, R^2^ = 0.91.

We also observed a peculiar solvent effect for O-CDs, which showed different emissive properties in different types of solvents. When 1.5 mg of O-CDs were dispersed in 10 mL of water, methanol, acetone, and tetrahydrofuran (THF) separately, the solution exhibited yellow, green, green and blue emission, respectively. (Figure 3a) This phenomenon is well known as solvatochromic shift in the emission spectrum caused by the solvent relaxation [30]. With the increase of polarity of solvent, there is an apparent red-shift according to the Lippert-Mataga equation [31,32]. A linear relationship between the solvent polarity and maximum emission wavelength of O-CDs was observed in Figure 3b.

**Figure 4:**
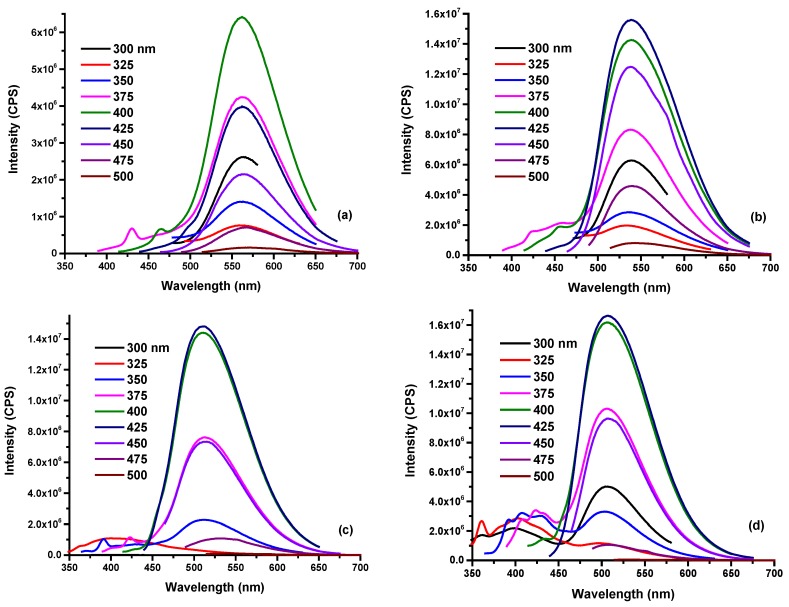
The fluorescence emission spectra of O-CDs dispersed in various solvents. (0.15 mg/mL) ((**a**) water, (**b**) methanol, (**c**) acetone and (**d**) tetrahydrofuran).

In Figure S1, we observed different solvents had no influence on the absorption peak (417 nm) of O-CDs in the low-energy region. However, unlike absorption results, the PL spectra varied significantly as a function of the polarity of solvent. At the same concentration, O-CDs exhibited lower emission intensity in water compared to dispersal in organic solvents, which was probably caused by higher dispersity in organic solvents due to the surface functional groups of O-CDs. (Figure 4) This phenomenon was confirmed by the fluorescence QY of O-CDs in different solvents, which were measured and summarized in Table S1. The QY was observed to be improved in organic solvents compared to in water. Furthermore, the excitation-independent PL gradually turned in to excitation-dependent PL with the decrease in the solvent polarity. Also, the maximum emission wavelength was 570, 539, 512 and 507 nm of O-CDs in water, methanol, acetone and THF, respectively, which corresponds to the varied emission light shown in Figure 3a.

### 3.3. Embedment of O-CDs into SPA

**Figure 5:**
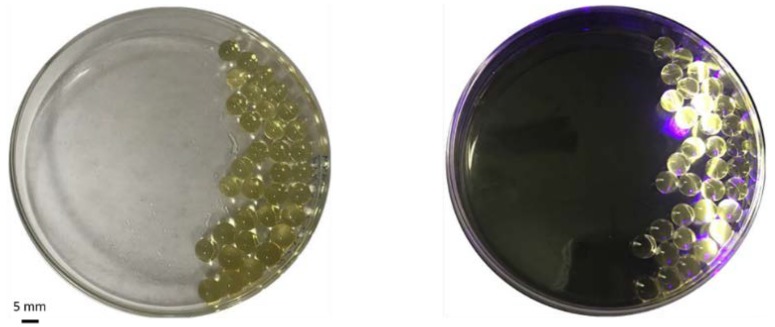
O-CDs embedded in sodium polyacrylate (SPA)-based beads under (**left**) regular light and (**right**) UV light.

As one type of superabsorbent, SPA has the greatest absorbency in water. However, the addition of inorganic salt or organic solvent will decrease the absorbency [33]. Therefore, to test if O-CDs can be absorbed by SPA, O-CDs aqueous solution was mixed with commercial Orbeez SPA-based beads. 200 µL of 0.5 mg/L O-CDs aqueous solution was mixed with 10 mg SPA-based beads. After one hour, the solution was completely absorbed into the beads and the beads exhibited orange PL under UV/vis light (405 ± 10 nm). (Figure 5) After natural desorption of water by exposing to the air over night, the orange beads’ PL remained, which indicated O-CDs were sequestered.

The prepared fluorescent polymer containing O-CDs allowed additive manufacturing of these materials which demands high amount of feedstock. 3D printing (a.k.a additive manufacturing) allows fabrication of objects to have complex geometries without retooling. To prepare the feedstock for the 3D printing process, O-CDs were mixed with SPA powder. It was crucial to find the ideal mass ratio of O-CDs and SPA mixture to minimize the number of O-CDs required for sufficient PL. It indicated in Figure S2a that when the ratio was 1:50, the mixture of CDs and SPA could not yield gelatinous form and the PL intensity revealed by the Figure S2b remained the same with the mass ratio decreased from 1:50 to 1:100. However, when the mass ratio decreased from 1:100 to 1:500, the PL intensity of the mixture decreased by 12.5% but remained a sufficient level. Therefore, 1:500 was selected as the optimal mass ratio for mixing CDs and SPA. After comparing the PL observation (Figure S2a) and spectra (Figure S2b) of O-CDs and SPA with different mass ratios (1:50, 1:100, 1:500), O-CDs and SPA were eventually mixed with a ratio of 1:500 by mass.

**Figure 6:**
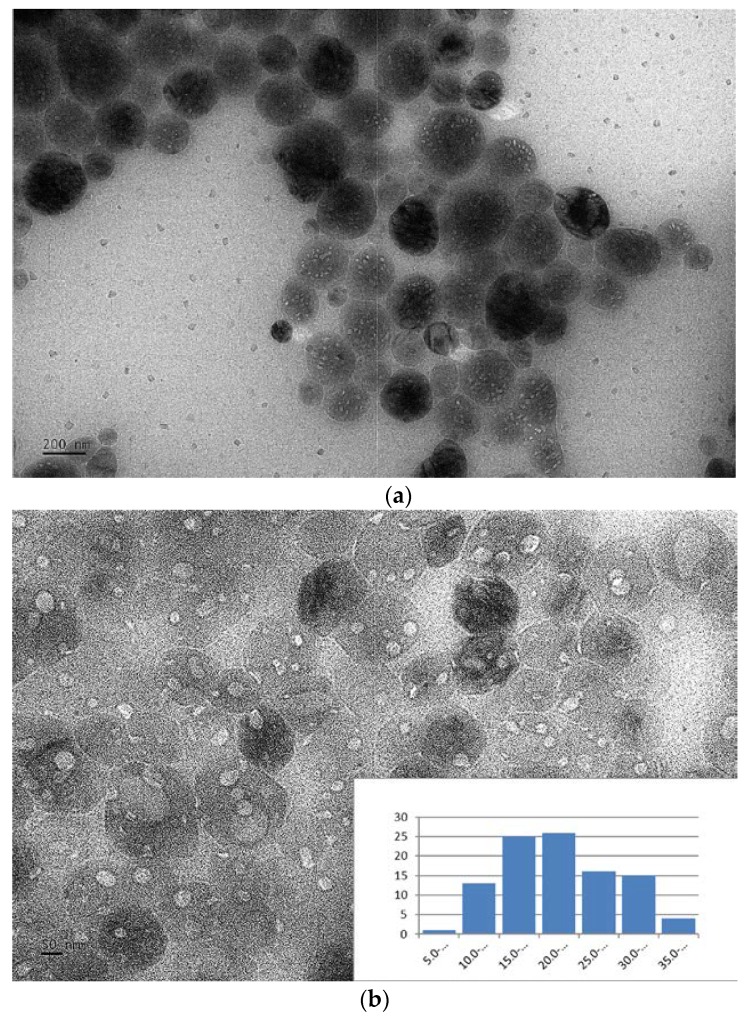
TEM image of O-CDs embedded in SPA. (**a**,**b**) are in different scale. The inset in (**b**) is the size histogram of O-CDs embedded in SPA.

Compared with the bare O-CDs and SPA, (Figure 1b and Figure S3a) the PL of O-CDs embedded in the SPA in solid state has been slightly changed. (Figure S3b) The emission peak position occurred red-shift from 570 to 608 nm, which provided an explanation of the orange PL of beads. However, we also observed the PL of O-CDs are still excitation-independent. Moreover, since O-CDs have the solvent effect, the same experiment has been performed with O-CDs after being embedded into the SPA to examine if the solvent effect still worked. The experimental result is shown in Figure S4. When the assembly was placed in water, SPA absorbed water along with the dispersion of O-CDs in water, which exhibited orange color. However, since organic solvents couldn’t enter or dissolve SPA, the solvent effect of O-CDs was not exhibited for O-CDs absorbed into the SPA. The TEM microscope recorded the images of O-CDs in the SPA (Figure 6a,b). Compared with the TEM image of bare SPA particles (Figure S5), the lighter colored spots show O-CDs with low-degree aggregation, which was previously confirmed by the low surface charge. Wee observe that even though O-CDs show a low degree of aggregation with an average size of 22 ± 7 nm (Figure 6b), they disperse well within the SPA particles, which explained the excellent PL in solid state.

**Figure 7:**
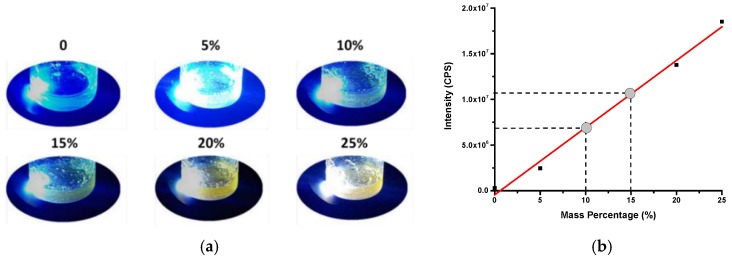
CDs-SPA mixed with the photopolymer resin with different mass percentage under UV lamp (365 nm) (**a**); The linear relation between the PL intensity (excitation: 425 nm; emission: 562 nm) and mass percentage of CDs-SPA in resin. R^2^ = 0.99 (**b**).

As for the PL effect of the 3D printed objective, we aimed to use highest amount of particles in the resin to maximize the fluorescence of the 3D printed component. Observed from Figure 7a, in order to exhibit the yellow PL of CDs-SPA, the minimum mass percentage should be 15%. In addition, fluorescence measurement in Figure 7b revealed the sufficient PL (>10^7^) could be achieved with the minimum mass percentage of 15%. Therefore, the minimum mass percentage of the mixture of CDs-SPA and resin to achieve the PL behavior was 15%. However, it is a limitation of the SLA printing process as described earlier [34] that higher content of additives results lack of photopolymerization of the clear resin we used for printing. We observed that for samples with above 25% volume fraction of CDs-SPA powder, curing and printing obtained were incomplete. Therefore, we limited our mixing amount to 25% with 150 mL resin filled inside the tank of the printer.

**Figure 8:**
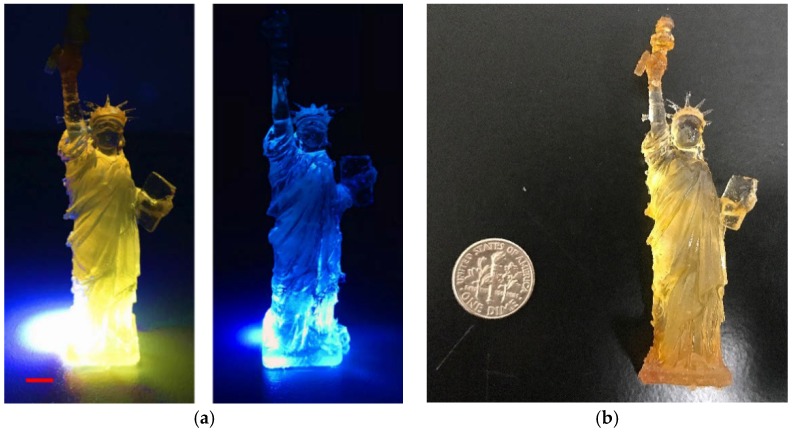
(**a**) 3D printing of the Statue of Liberty using (**left**) O-CDs and (**right**) control. (The bar is 5 cm); (**b**) The comparison of the specimen with a US dime.

With the CDs-SPA composite as the ink, Figure 8a shows an example specimen, the statue of liberty, and the specimen control printed without O-CDs using the same stereolithography file (Figure S6). The control experiment was carried out by mixing SPA without embedded O-CDs with the commercial photopolymer in the same ratio by mass as the experimental counterpart. In comparison, due to the presence of well-dispersed O-CDs, the liberty statue specimen exhibited bright orange fluorescence while the control scattered the blue color of UV/vis light. Also, the specimens were compared with a US dime in Figure 8b, which could illustrate the actual size of the printed specimens.

**Figure 9:**
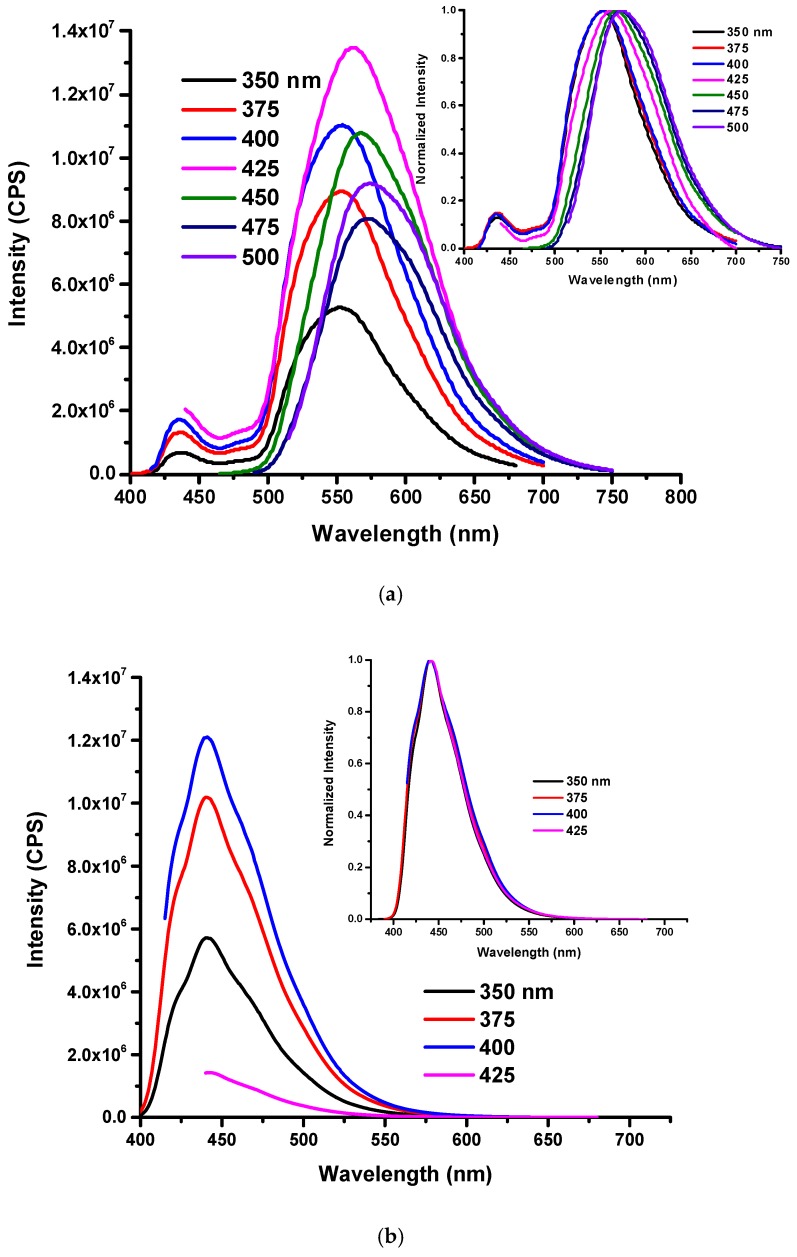
Fluorescence measurement of (**a**) the mixture of O-CDs-SPA in the photopolymer resin with a mass percentage of 25% and (**b**) photopolymer resin.

In addition, the fluorescence measurement of the O-CDs-SPA-photopolymer in solid state (Figure 9a) showed us the mixture exhibited an excitation-dependent emission with the maximum excitation and emission wavelengths as 425 and 562 nm, respectively. Meanwhile, the photopolymer resin showed the maximum excitation and emission wavelengths at 400 and 440 nm, respectively. Also, the comparison between Figure 9a,b independently showed the peaks of O-CDs-SPA and the resin, which indicated the little interference of the photopolymer to the PL behavior of O-CDs-SPA benefited from O-CDs with a long emission wavelength.

## 4. Conclusions

In conclusion, SPA and CDs have been used in 3D printing, in conjunction with a photopolymer, for the first time to create a photoluminescent material. O-CDs could be synthesized via a fast ultrasonication approach with low energy consumption. The synthesized O-CDs were small (~2 nm) and exhibited yellow PL with an excitation-independent but solvent dependent behavior. The special PL property contributed to the potential of O-CDs as a potential photoluminescent 3D printing material by embedding O-CDs into a superabsorbent polymer-SPA. The embedment prevented the high degree of self-aggregation and achieves the PL of O-CDs in solid state. Also, it did not change the PL behavior of O-CDs. Even coated with photopolymer resin, the CDs-SPA still possess long emission wavelength, which reduced the interreference from light source and resin. Therefore, O-CDs may be applied to future 3D printing of many designs and industrial products such as UV light converters, sunglasses and probes for environment, biological or medical purposes, which benefits from their PL properties and small sizes.

## Supplementary Materials

The following are available online at http://www.mdpi.com/2073-4360/10/8/921/s1, Figure S1: The UV/vis absorption spectra of O-CDs dispersed in various solvents, Figure S2: O-CDs in aqueous medium mixed with SPA with different mass ratio (1:500, 1:100 and 1:50, from left to right) before and after the evaporation of water; The fluorescence spectra of O-CDs mixed with SPA with different ratios under an excitation wavelength of 400 nm, Figure S3: The fluorescence emission spectrum of SPA powder and O-CDs embedded in SPA, Figure S4: O-CDs embedded in SPA in different solvents (4 mg/mL). (From left to right: water, methanol, acetone, THF), Figure S5: TEM image of SPA particles alone, Figure S6: The stereolithography file for printing statue of liberty, Table S1: The fluorescence quantum yield of the O-CDs dispersed in different solvents.
