# Supplementary material for: Embedding Carbon Dots in Superabsorbent Polymers for Additive Manufacturing
Source: Polymers (Basel). 2018 Aug 17;10(8):921. doi: 10.3390/polym10080921 (PMC6403561; doi:10.3390/polym10080921)
Supplement: Supplementary file 1 [file polymers-10-00921-s001.docx]

Supplementary Materials: Embedding Carbon Dots in Superabsorbent Polymers for Additive Manufacturing

Yiqun Zhou, Keenan J. Mintz, Cagri Y. Oztan, Sajini D. Hettiarachchi, Zhili Peng, Elif S. Seven, Piumi Y. Liyanage, Sabrina De La Torre, Emrah Celik, Roger M. Leblanc

**1.** The data sheet of the commercial FLGPCL02 photopolymer resin containing its physical properties is linked to <https://formlabs.com/media/upload/Clear-DataSheet.pdf>

**2.** The data sheet of Orbeez commercial SPA-based beads is linked to https://www.toysrus.com/buy/arts-crafts/orbeez-ultimate-soothing-spa-47216-70135156

**3.** Quantum yield measurement

The quantum yield calculation was based on the equation [1].


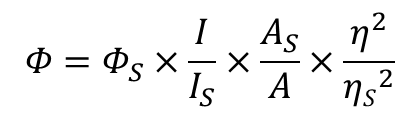


where Φ_s_ is the quantum yield of the standard solution, I is the integrated area under the fluorescence emission peak, A is the absorbance of the solution at the excitation wavelength and η is the average refractive index of the solution. Subscripts S refer to the standard solution.

The UV/vis absorption and fluorescence emission spectra were recorded for O-CDs in different solvents and standard (tris(bipyridine)ruthenium(II) chloride dissolved in H_2_O. Rhodamine 6g dissolved in ethanol was used as another standard to confirm the quantum yields. The Φ_R_ of tris(bipyridine)ruthenium(II) chloride in H_2_O under 436 nm was 2.8 % [2] and it was 94 % of rhodamine 6g in ethanol under 488 nm [3]. The η of water, methanol, acetone and THF are 1.333, 1.331, 1.360 and 1.407, respectively.

**4.** Parameters of 3D printing

The resolution of the printer was determined by hardware and software printing parameters. Hardware parameters determining the printing resolution were the laser spot size and the resolution of the actuators controlling the dimensional accuracy. Laser spot size was 155 microns and layer resolution could be as low as 25 microns. The laser is EN 60825-1:2007 certified, Class 1 Laser Product with a wavelength of 405nm emitting in violet color. Laser power is 250mW. Preform software was used to control slicing and other printing parameters such as speed, laser energy, etc. Although laser spot size limited the minimum feature size, we could not achieve that resolution using carbon dot powder <600 microns. Smaller sized powders must be used to achieve maximum resolution of the prints.

**

**Figure S1.** The UV/vis absorption spectra of O-CDs dispersed in various solvents.

| 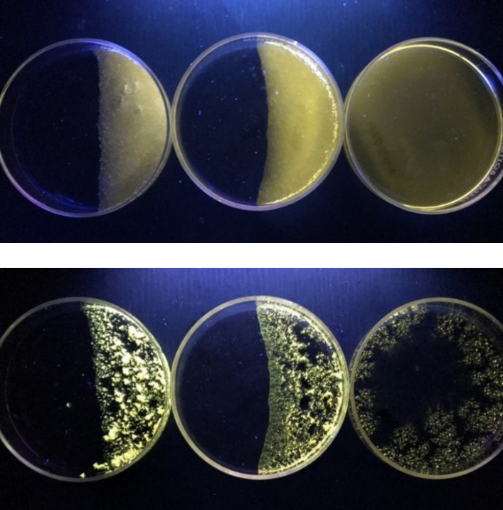 |  |
| --- | --- |
| (**a**) | (**b**) |

**Figure S2.** O-CDs in aqueous medium mixed with SPA with different mass ratio (1:500, 1:100 and 1:50, from left to right) before (upper) and after (lower) the evaporation of water **(a)**; The fluorescence spectra of O-CDs mixed with SPA with different ratios under an excitation wavelength of 400 nm **(b)**.

**(a)**

**(b)**

**Figure S3**. The fluorescence emission spectrum of SPA powder **(a)** and O-CDs embedded in SPA **(b)**. (Inset is the normalized spectrum)


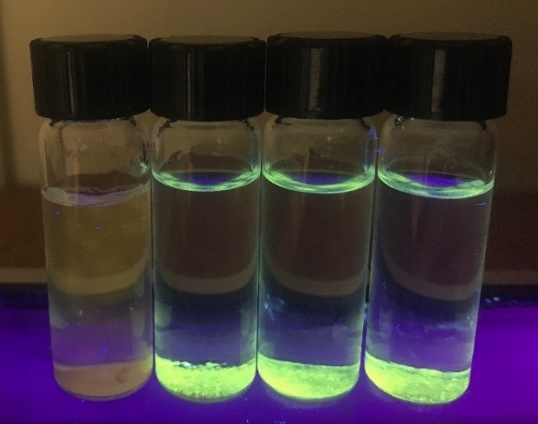


**Figure S4.** O-CDs embedded in SPA in different solvents (4 mg/mL). (From left to right: water, methanol, acetone, THF).


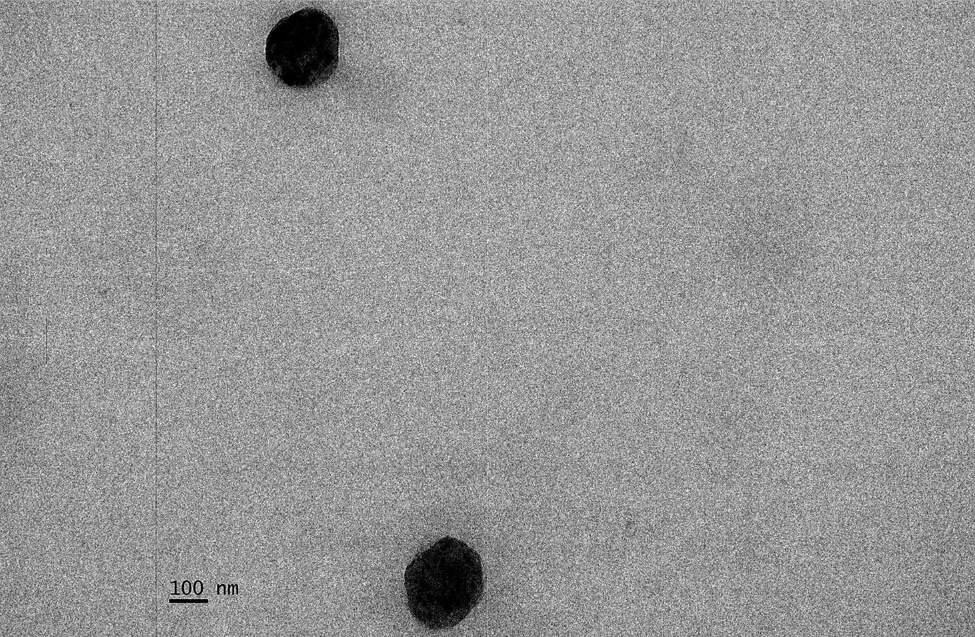


**Figure S5.** TEM image of SPA particles alone.

**
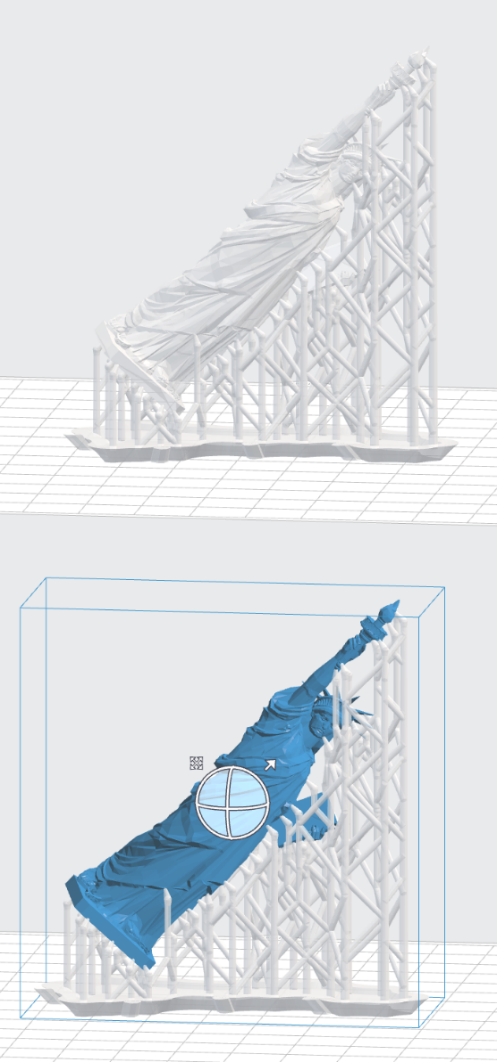
**

**Figure S6.** The stereolithography file for printing statue of liberty.

**Table S1.** The fluorescence quantum yield of the O-CDs dispersed in different solvents.

|  | Water | Methanol | Acetone | THF |
| --- | --- | --- | --- | --- |
| Quantum yield | 1 % | 1 % | 7 % | 2 % |

**References**

1. Williams, A.T.R.; Winfield, S.A.; Miller, J.N. Relative fluorescence quantum yields using a computer-controlled luminescence spectrometer. *Analyst* **1983**, *108*, 1067–1071.

2. Katsumi, N. Synthesis, luminescence quantum yields, and lifetimes of trischelated ruthenium(ii) mixed-ligand complexes including 3,3′-dimethyl-2,2′-bipyridyl. *Bull. Chem. Soc. Jpn.* **1982**, *55*, 2697–2705.

3. Douglas, M.; E., R.G.; G., S.P. Solvent dependence of the fluorescence lifetimes of xanthene dyes. *Photochem. Photobiol.* **1999**, *70*, 737–744.
